# Case of Morbid Adhesion of the Placenta

**Published:** 1835-07-01

**Authors:** 


### *Case of Morbid Adhesion of the Placenta. By* Dr. Litchfield.

Mary Farrell, ætat. 32, was attended in her first confinement by Mr. Barry, of Judd street, Brunswick square, in March last.

The labour, as described by Mr. Barry, was very lingering, the pains slight and at long intervals. Sixteen hours after the commencement of labour, the accoucheur in attendance administered half a drachm of the powdered ergot of rye, and this dose was repeated every two hours; at the expiration of twenty-four hours, a dead child was expelled, and the uterus contracted forcibly round the placenta, so as to baffle the repeated efforts of the accoucheur to remove it.

On the morning of the 12th of March, eighteen hours after the delivery of the patient, I was requested to see her in consultation. The uterus was found, upon examination, high up beneath the abdominal parietes, and contracted at its fundus into a hard and irregular tumour. The external parts of generation were swollen and painful, and the os uteri so rigid and unyielding as to resist the persevering efforts of the hand to dilate it and reach the placenta.

The pulse at this period was full, hard, and at ninety-five; the tongue furred and feverish, the face flushed, and the patient complained of severe pain in the head; to relieve these symptoms, and lessen the force of the muscular contractions, I ordered ten ounces of blood to be taken from the arm, and prescribed small repeated doses of tartarized antimony; fomentations with flannels were also applied freely to the swollen pudenda; under this treatment the violence of the symptoms subsided, and fresh and long-continued, but unsuccessful attempts were again made, to dilate the os uteri, and detach the placenta.

During the latter months of pregnancy the patient had complained of fixed pain in the womb, arising, as she believed, from a blow in the abdomen; it seemed probable, under these circumstances, that the vessels of the uterine structure being stimulated to undue action, had thrown out coagulable lymph, by which the placental and uterine surfaces had become morbidly united. Being of opinion that it would be impossible, in the present state of the parts, to reach and overcome this adhesion, and having no fear of immediate hæmorrhage, I resolved to wait, and watch closely both the local and constitutional symptoms, abstaining for the present from further manual interference.

In this way the case continued to progress until the fourth day, the patient remaining in a very satisfactory state. On the fourth day after delivery the discharge, which had set-in as usual, became more copious in quantity, of a green colour, and very offensive smell: this last character was in some degree corrected by the use of injections of chloride of soda, and the patient went on, without any unfavourable symptom, till the ninth day, when a portion of the placental mass, equal to about one third of its usual weight, was thrown off in a state of putridity. From this period, small portions of the placenta continued to detach themselves at intervals, until the twenty-first day, when all that remained of the adherent structure was thrown off.

The progress of the case was unattended with pain or hæmorrhage; the patient improved rapidly during the time in spirits, strength, and appetite, and, at the end of a month from her delivery, menstruated in a regular way. Strong cartilaginous bands were found in the placental mass.

